# Loss of *fuss* in *Drosophila melanogaster* results in decreased locomotor activity due to an increased number of pauses

**DOI:** 10.17912/micropub.biology.000230

**Published:** 2020-03-09

**Authors:** Mathias Rass, Svenja Oestreich, Ardi Manaj, Stephan Schneuwly

**Affiliations:** 1 Department of Developmental Biology, Institute of Zoology, University of Regensburg, Regensburg, Bavaria, Germany

**Figure 1. Loss of fuss results in reduced locomotor activity:**
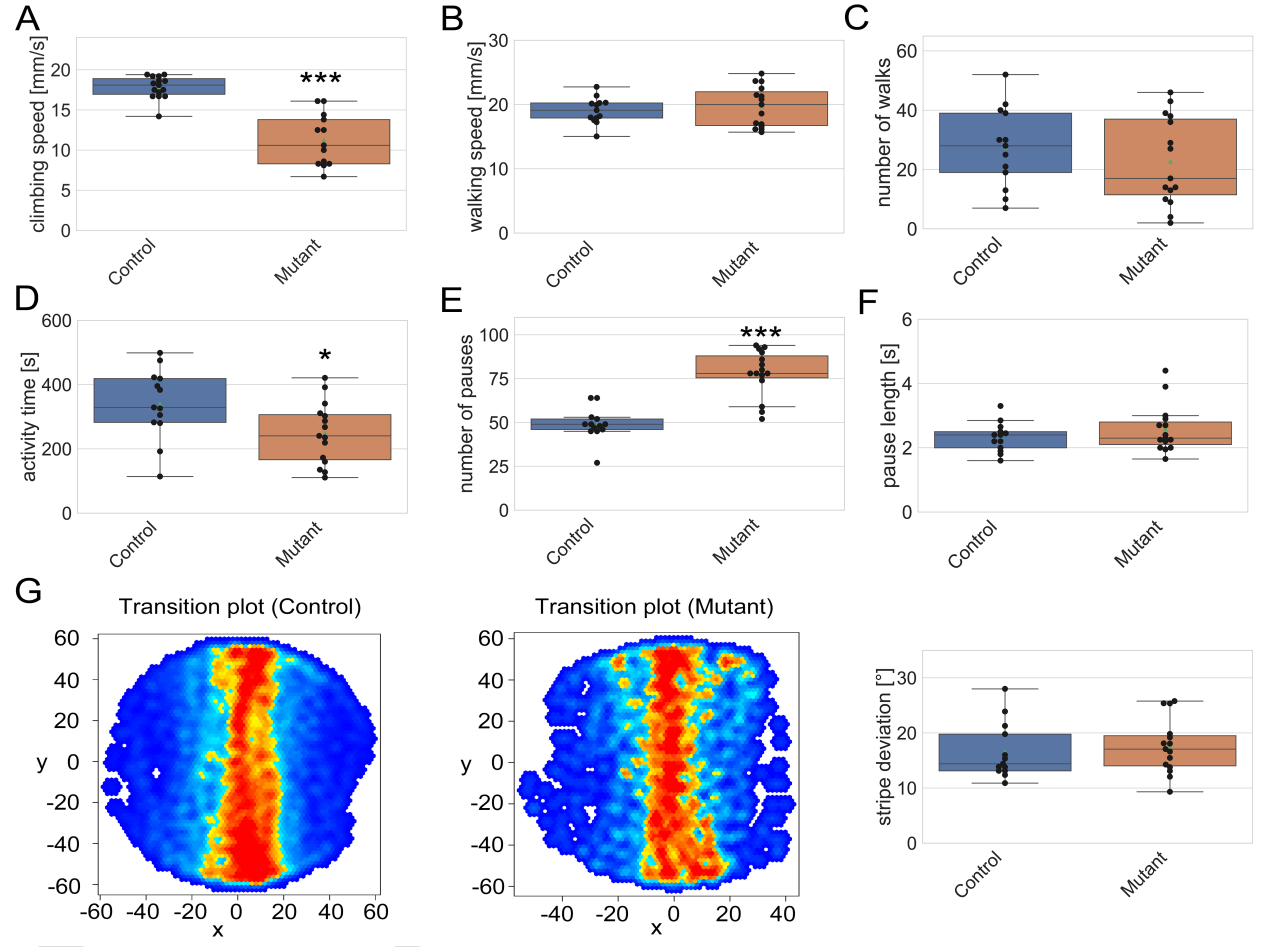
(A) Climbing speed of homozygous *fuss* mutant flies is reduced in contrast to control flies (mean speed (Mutant): 11.2 mm/s; mean speed (Control): 17.8 mm/s; Student’s t-test: p < 0.001). (B) In a Buridan’s assay, walking speed during active times is similar between genotypes (mean speed (Mutant): 19.7 mm/s, mean speed (Control): 19.1 mm/s). (C) Number of walks between stripes is slightly but insignificantly reduced in *fuss* mutant flies compared to controls (mean number of walks (Mutant): 22.7; mean number of walks (Control): 27.4). (D) Activity time is significantly reduced in *fuss* mutant flies compared to controls (mean activity time (Mutant): 248.1 s; mean activity time (Control): 340.3 s; Student’s t-test: p < 0.05). (E) Number of pauses is strongly increased in *fuss* mutant flies (mean number of pauses (Mutant): 78.0; mean number of pauses (Control): 49.1; Student’s t-test: p < 0.001). (F) Pause length is similar between *fuss* mutant flies and controls (mean pause length (Mutant): 2.6s; mean pause length (Control): 2.3s). (G) Stripe perception of *fuss* mutant flies is similar to control flies as shown by transition plots and stripe deviation (mean stripe deviation (Mutant): 17.6°; mean stripe deviation (Control): 16.6°). n = 13 – 15 for each genotype.

## Description

In *Drosophila melanogaster*, two members of the Ski/Sno protein family exist, the Ski novel oncogene (Snoo) (Barrio *et al.* 2007) and the functional Smad suppressing element (Fuss) (Fischer *et al.* 2012). We have previously shown that Fuss is specifically expressed in gustatory receptor neurons (GRNs) and interneurons in the central nervous system (CNS). We created a loss of function allele *fuss^delDS^* and showed that loss of *fuss* had no impact on survival rate during development or on adult life span. In fact, *fuss^delDS^* flies display an impaired bitter GRN development and bitter taste reception (Rass *et al.* 2019).

To address if *fuss^delDS^* shows other behavioural phenotypes we tested these flies in different behavioural assays (circadian rhythm, optomotor response, climbing and Buridan’s paradigm), but we only found phenotypes in a climbing (Botella *et al.* 2004) and Buridan’s paradigm assay (Bülthoff *et al.* 1982; Colomb *et al.* 2012). In the climbing assay, flies were allowed to climb as high as possible in a pipette within 12 seconds. Interestingly, although *fuss* is not expressed in motoneurons, a strong reduction of the mean climbing speed of homozygous mutant *fuss^delDS^* flies was detected if compared to heterozygous controls (*fuss^delDS^* /+) (Fig 1, A). Upon closer examination of this phenotype, we noticed that the flies did not show any impairment in climbing capability but rather exhibited an increased number of pauses during testing. To record several different traits, we chose to test the flies using the Buridan’s paradigm assay. One day old flies’ wings were clipped, flies were allowed to recover for one night and underwent testing the next day. Flies were placed on a platform surrounded by water. The arena is homogenously illuminated except for two black stripes on opposite sides. If flies perceive the stripes, they will try to reach them mainly in an alternating fashion. The movement of every fly was recorded for 10 minutes with BuriTrack and analysed with the CeTrAn analysis software (Colomb *et al.* 2012). Interestingly, no difference between mutant and control flies in speed could be observed anymore, because the speed in this assay is only measured during activity (Fig 1, B). Furthermore, the number of walks between stripes was not significantly different between genotypes and showed only a slight tendency to a lesser number of walks between stripes in mutant flies (Fig 1, C). However, the overall activity time of mutant flies was significantly reduced in contrast to control flies (Fig 1, D). Similar to our previous observation, the number of pauses was strongly increased in mutant flies (Fig 1, E), whereas the pause length was unchanged (Fig 1, F). Overall, we can say that, due to the increased number of parameters recorded via BuriTrack and analysed with CeTrAn, the reduced climbing speed observed in the climbing assay is not the consequence of slower walking, but rather due to an increased number of pauses during walking, which ultimately leads to a reduced locomotor activity. Additionally, we have found that mutant *fuss^delDS ^*flies have no problems in perceiving the black stripes as shown by the average trajectory plot and the stripe deviation (Fig 1, G).

In future experiments, it will be of particular interest how the loss of *fuss* impairs the interneurons on a molecular and cellular level and if these interneurons are somehow synaptically connected to motoneurons, which would explain the increased number of walking pauses. In humans a reduced expression of the *fuss* homolog *Skor1* has been shown to be linked to Restless Legs Syndrome (RLS) (Catoire *et al.* 2018). However, it is rather unlikely that fly *fuss* has a similar function as human *Skor1*, or that *fuss* mutant flies could serve as a model for RLS, because a loss of function *dBTBD9* RLS fly model is associated with a higher activity and a reduced number of pauses in Buridan’s assay (Freeman *et al.* 2012).

## Methods

Generation and characterisation of the *fuss^delDS ^*(FBal0349586) allele has been described before (Rass *et al.* 2019). *The fuss^delDS ^*allele has been introduced into wild-type Berlin (WTB) background by backcrossing it five times to WTB flies. In the behavioural assays, only male flies underwent testing and homozygous *fuss^delDS^* flies were crossed to WTB and heterozygous progeny served as controls. *fuss^delDS^/+* control flies did not show differences compared to WTB males. In climbing and Buridan´s assays, thirteen control flies and fifteen mutant flies have been analysed. For Buridan´s assay, we used the devices and software described previously in Colomb *et al.* (2012). For data visualisation the Python libraries pandas, matplotlib and seaborn have been used. For statistical testing the Python library SciPy was used.
